# Neutrophils in community-acquired pneumonia: parallels in dysfunction at the extremes of age

**DOI:** 10.1136/thoraxjnl-2018-212826

**Published:** 2019-11-15

**Authors:** Frances Susanna Grudzinska, Malcolm Brodlie, Barnaby R Scholefield, Thomas Jackson, Aaron Scott, David R Thickett, Elizabeth Sapey

**Affiliations:** 1 Institute of Inflammation and Ageing, University of Birmingham College of Medical and Dental Sciences, Birmingham, UK; 2 Institute of Cellular Medicine, Newcastle University, Newcastle upon Tyne, UK

**Keywords:** pneumonia, neutrophil biology

## Abstract

"Science means constantly walking a tight rope" Heinrich Rohrer, physicist, 1933. Community-acquired pneumonia (CAP) is the leading cause of death from infectious disease worldwide and disproportionately affects older adults and children. In high-income countries, pneumonia is one of the most common reasons for hospitalisation and (when recurrent) is associated with a risk of developing chronic pulmonary conditions in adulthood. Pneumococcal pneumonia is particularly prevalent in older adults, and here, pneumonia is still associated with significant mortality despite the widespread use of pneumococcal vaccination in middleand high-income countries and a low prevalence of resistant organisms. In older adults, 11% of pneumonia survivors are readmitted within months of discharge, often with a further pneumonia episode and with worse outcomes. In children, recurrent pneumonia occurs in approximately 10% of survivors and therefore is a significant cause of healthcare use. Current antibiotic trials focus on short-term outcomes and increasingly shorter courses of antibiotic therapy. However, the high requirement for further treatment for recurrent pneumonia questions the effectiveness of current strategies, and there is increasing global concern about our reliance on antibiotics to treat infections. Novel therapeutic targets and approaches are needed to improve outcomes. Neutrophils are the most abundant immune cell and among the first responders to infection. Appropriate neutrophil responses are crucial to host defence, as evidenced by the poor outcomes seen in neutropenia. Neutrophils from older adults appear to be dysfunctional, displaying a reduced ability to target infected or inflamed tissue, poor phagocytic responses and a reduced capacity to release neutrophil extracellular traps (NETs); this occurs in health, but responses are further diminished during infection and particularly during sepsis, where a reduced response to granulocyte colony-stimulating factor (G-CSF) inhibits the release of immature neutrophils from the bone marrow. Of note, neutrophil responses are similar in preterm infants. Here, the storage pool is decreased, neutrophils are less able to degranulate, have a reduced migratory capacity and are less able to release NETs. Less is known about neutrophil function from older children, but theoretically, impaired functions might increase susceptibility to infections. Targeting these blunted responses may offer a new paradigm for treating CAP, but modifying neutrophil behaviour is challenging; reducing their numbers or inhibiting their function is associated with poor clinical outcomes from infection. Uncontrolled activation and degranulation can cause significant host tissue damage. Any neutrophil-based intervention must walk the tightrope described by Heinrich Rohrer, facilitating necessary phagocytic functions while preventing bystander host damage, and this is a significant challenge which this review will explore.

## INTRODUCTION

Community-acquired pneumonia (CAP) is the leading cause of infectious death globally^1 2^ and disproportionately affects those at the extremes of age. CAP is one of the leading causes of sepsis in children (we have defined preterm infants as infants born before 38 weeks’ gestation; infants as term to 1 year of age; children as 1–12 years of age; and young adults as 12–18 years of age), and pneumonia has some of the poorest outcomes in childhood sepsis.^3^ Peak incidence of CAP in children occurs at 2 years of age.^2^ At the other end of the spectrum, more than 80% of all episodes of CAP occur after 60 years of age, where CAP-related mortality is highest.^4^ Older adults with CAP are more likely to develop serious complications such as acute respiratory distress syndrome (ARDS) and sepsis.^5^

In child survivors of sepsis, a quarter of children are discharged with some form of disability, and 1 in 10 ha severe disability at discharge.^3^ Recurrent pneumonia occurs in up to 10% of CAP cases in children. Adult survivors of CAP are at increased risk of death for up to 10 years following recovery, and CAP survivorship is associated with poorer life satisfaction, increased healthcare expenditure, need for domiciliary care, frailty^5^ and high readmission rates.^6^ Eleven per cent of older adults are readmitted within 30 days, often for a secondary infection.^6^ These secondary infections are associated with increased length of stay, mortality and further readmissions.^6^

The direct and indirect costs of pneumonia exceed €10 billion per annum in Europe,^7^ representing a substantial cause of disability adjusted life years lost.^7^ Although the prevalence of pneumonia in young children is falling (in part due to widespread childhood vaccination programmes), it remains a significant cause of mortality and morbidity worldwide.^2^ Furthermore, the United Nations predict a doubling in the number of people aged over 65 years in the next two decades,^8^ and therefore, CAP is likely to remain a significant clinical and economic burden globally at both extremes of age. Antibiotics, intravenous fluids and oxygen remain cornerstones of CAP treatment. However, while improving CAP management to improve health outcomes is significant to patients, carers, healthcare providers and policy makers, this must be balanced with the need for appropriate antibiotic stewardship.

This review will explore why children and older adults are more susceptible to pneumonia and consider what therapeutic strategies might be deployed to improve outcomes.

## Aetiology of CAP in adults

Common causes of CAP are listed in table 1, although identifying a causative pathogen is challenging, even in prospective studies.^4^

**Table 1:** Aetiology of hospitalised CAP in adults in Europe

Causative pathogen	Frequency (%)
Bacterial pathogens	0–47
*Streptococcus pneumoniae*	11–68
*Haemophilus influenzae*	5.3–12.3
*Legionella pneumophilia*	0–12.8
*Staphylococcus aureus*	0–11.8
*Moraxella catarrhalis*	0–5.4
Gram-negative bacteria	0–24.2
*Mycoplasma pneumoniae*	0.7–32.0
*Chlamydia pneumoniae*	1.0–26.5
Viruses	0–34
Influenza viruses	15–19
Rhinoviruses	0.7–12.0
Respiratory syncytial virus	3–4
Parainfluenza	3
Metapneumovirus	3
Adenoviruses	0.4–04
Bacterial–viral coinfection	31

The main causative organisms for CAP are listed, with frequency expressed as a percentage. Relative frequencies are pooled from multiple studies; some studies did not test for certain pathogens. Adapted from Welte and Köhnlein,^4^ Ewig *et al* 9 and Holter *et al*.^10^

CAP, community-acquired pneumonia.

Across high-income countries, *Streptococcus pneumoniae* (SP), non-typeable *Haemophilus influenzae* (ntHI) and *Mycoplasma pneumoniae* are the most common causative bacteria identified in CAP,^4 9^ with no significant differences in unselected cohorts of older versus younger adults. Certain patient characteristics increase the likelihood of different causative bacteria. Gram-negative pathogens, ntHI and *Staphylococcus aureus* are more commonly found in patients with existing lung disease and those from nursing homes^4^ who have significantly increased mortality from pneumonia. Bacterial and viral coinfections are common, identified in up to 31% of adults admitted to hospital with CAP; however, pure viral CAP appears to be less common than CAP with a pure bacterial cause.^10^ Secondary bacterial pneumonia following viral infection is associated with high mortality and is the leading cause of death from influenza.^11^

## Aetiology of CAP in children

Determining aetiology is more challenging in children than in adults. Young children are not typically able to expectorate sputum and have low rates of blood culture positivity. Children also have high carriage or colonisation rates of common respiratory pathogens. For example, in healthy children, certain pathogens can be present at rates of 20%–25% in nasopharyngeal swabs^12 13^; however, certain pathogens are infrequently detected in asymptomatic children, and the presence of these usually indicates clinically relevant infection. Overall, viral pathogens are more common in children; common causes are listed in table 2. Bacterial–viral coinfection is also common^12^ and is associated with increased risk of adverse outcomes as reported in adult populations.

**Table 2:** The aetiology of CAP requiring hospitalisation in children across Europe

Pathogen	Frequency (%)
Respiratory syncytial virus	20–28
Rhinovirus	15–27
Human metapneumovirus	10–13
Adenovirus	4.3–27.0
*Mycoplasma pneumoniae*	8.0–8.2
Parainfluenza viruses	4.7–7.0
Influenza viruses	6.9–7.0
*Streptococcus pneumoniae*	4.0–25.3
*Haemophilus influenza* *e*	32.6
*Moraxella catarrhalis*	44.7

The main causative organisms for CAP are listed, with frequency expressed as a percentage. Adapted from Bhuiyan *et al* 12 and Jain *et al*.^13^

## Risk factors for developing CAP in older adults and young children

**Figure 1:**
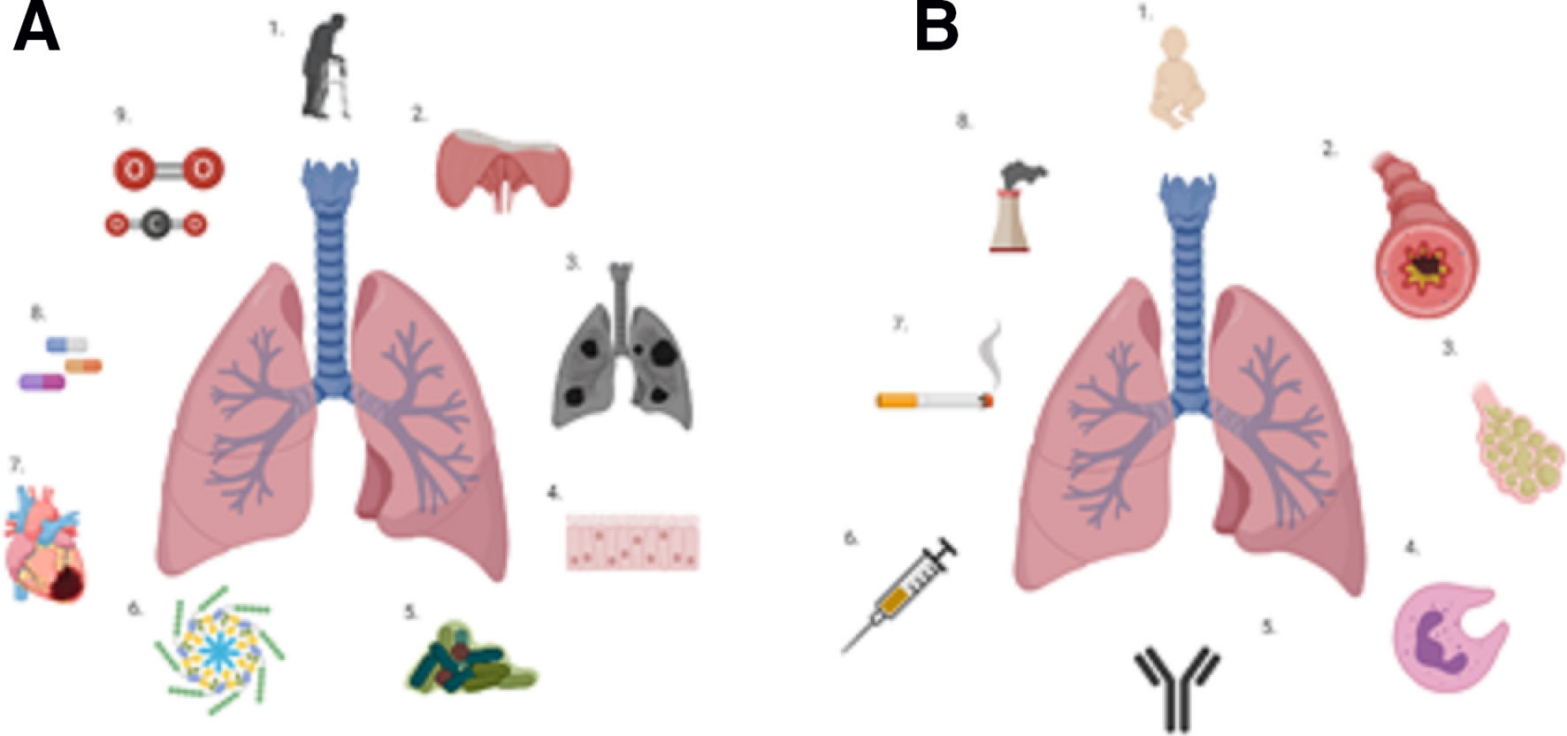
Factors increasing susceptibility to CAP. (A) Factors present in older adults (starting at the top and moving clockwise): (1) age alone is associated with an increased burden of CAP; (2) the mechanics of ventilation are impaired with age, the thoracic cage is less compliant, the diaphragm is weaker, and microaspiration is common; (3) the lung parenchyma loses elasticity, leading to senile emphysema; (4) the mucociliary escalator is less efficient in older adults, reducing the clearance of bacteria and microparticles from the lung; (5) the lung microbiome alters with age^84^; (6) ageing is associated with a low-grade pulmonary inflammation; (7) multimorbidity and poor nutritional status; (8) polypharmacy are common in old age and CAP; and (9) older adults exhibit reduced responsiveness to hypoxia and hypercapnia.^85^ (B) Factors present in younger children (starting at the top and moving clockwise): (1) younger age is associated with risk of CAP^86^; (2) asthma increases the risk of CAP^87^; (3) previous respiratory infection increases the risk of future CAP^86^; (4) impaired innate immunity; (5) impaired adaptive immunity increases the risk of developing CAP; (6) not being vaccinated against common respiratory pathogens increases the risk of CAP; (7) passive smoking increases the risk of CAP; and (8) environmental pollution increases the risk of CAP in children. CAP, community-acquired pneumonia.

There are some well-established risk factors that partially explain the high incidence of CAP in the older adult. Advanced age alone is a significant risk factor for CAP,^9^ and during ageing, the human host and respiratory system undergo structural, physiological and pathological changes that can lower resilience to infection, as described in figure 1A. These often reflect the accumulation of multimorbidity and organ insults endured over the years. Older adults are often more challenging to diagnose with CAP. They commonly present late with atypical features, such as delirium while lacking classical signs and symptoms of pneumonia, such as fever and cough.^14^

Vaccination against pneumococcal disease is common in high-income countries.^15^ In older adults, especially frail older adults, the response to vaccination is impaired.^16^ In older adults, antibody levels often drop below protective levels 5 years after vaccination.^17^

Young children have not had the lifespan to develop accumulated insults, which increase the susceptibility to infection. However, there are accepted risk factors, including younger age, existing respiratory disease, previous respiratory infection, vaccination status and presence of environmental pollution, including parental smoking (see figure 1B).

## Host immune system at the extremes of age

The host immune response to infection changes across the life course, both in the very young and with age and frailty. Changes associated with increasing age are termed immunosenescence. The deficits in innate and adaptive immunity seen with ageing are well described, and all predispose towards a less organised and effective response to infection, as described in table 3. Studies in children have mainly focused on those with the presence of a specific disease or immunodeficiency, but in the few studies of healthy children, there also appear to be alterations in immune function, which might increase the susceptibility to infection.

**Table 3:** Alterations in the innate and adaptive immune system of infants and older adults

Cell	Changes in healthy older adults	Changes seen in healthy infants
Neutrophils	Increased incidence of neutropenia^32^ Altered cytokine production^34^ Impaired migration^33^ Reduced pathogen killing^35^ Increased apoptosis^38^	Reduced migratory ability^55^ Reduced degranulation^57^ Preserved phagocytosis^88^ Reduced NET generation^60^ Preserved ROS generation^53^
Macrophage/ monocytes	Reduced phagocytosis and production of free radicals^89^ Possible reduced efferocytosis^90^ Decreased ability to antigen present due to reduced expression of MHC class II^91^	Reduced ability to secrete inflammatory mediators after LPS stimulation^92^
Dendritic cells	Relative frequency controversial^93^ DC function maintained in healthy older adults^94^ but impaired in frail older adults^95^	Negative correlation between the number of plasmacytoid DC and age
NK cells	Increased numbers but reduced cytotoxicity^96^	Reduced cytotoxicity^97^ NK cells from children are phenotypically different from adults in terms of cell surface receptors.^98^
Adaptive Immunity	Reduced numbers of naïve T cells T cell exhaustion Decreased capacity to respond to novel antigens Lower affinity antibodies Reduced numbers of B cells^99^	Increased Tregs Blunted humoral responses^97^

Table 3 gives an overview of cellular features of changing features of immunity in humans with ages. Features in neutrophils are expanded on later in the text.

DC, dendritic cell; LPS, Lipopolysaccaride; MHC, major histocompatibility complex; NET, neutrophil extracellular trap; NK, natural killer; ROS, reactive oxygen species; Treg, T regulatory lymphocyte.

The immune system in neonates must strike a balance between exposure to multiple pathogens while tolerating acquisition of colonising organisms, without creating a hostile inflammatory environment. Neonates rely heavily on transfer of immunoglobulins from the mother both via the placenta and in breastmilk; thus, maternal immunity is key to early protective responses.^18^ Mode of delivery also influences the early development of immunity in the neonate.

Alveolar macrophages (AMs) are the first cells activated by pulmonary infection and are avid phagocytes^19^; their response is especially important in SP pneumonia. Once their phagocytic capacity is overwhelmed, they orchestrate a proinflammatory, antimicrobial local environment to facilitate pathogen killing by recruiting neutrophils to the airways. AMs are also able to coordinate an anti-inflammatory and restorative environment to facilitate repair, clearing apoptotic neutrophils to allow resolution of inflammation and prevent excessive tissue damage from the cytotoxic contents of neutrophil granules.^19^

Neutrophils are key effector cells during infections in CAP, as shown by the high incidence and severity of CAP in patients with specific neutrophil deficits^20^ and in animal models of neutrophil depletion, which demonstrate increased susceptibility and severity of pneumonia.^21^ Neutropenia is an important risk factor for CAP; however, in this population, the aetiology is often due to Gram-negative pathogens.^22^ There have been advances in our understanding of how neutrophil functions alter with age and infection, which may increase the susceptibility to CAP.

### Classical neutrophil responses to bacterial and viral challenges

**Figure 2:**
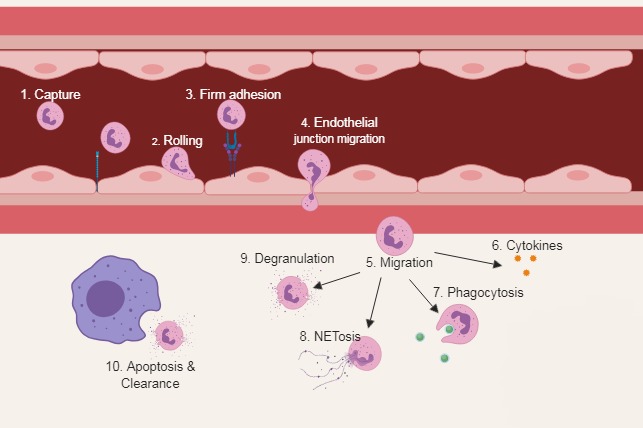
Classical neutrophil functions. (1) Neutrophils actively patrol the circulation and are able to detect host-derived and pathogen-derived inflammatory signals via interaction with endothelial cells. (2) Neutrophils become tethered and roll along the endothelial wall in a process mediated by selectins. (3) Activation of integrins causes firm adhesion. (4) Cytoskeletal rearrangement allows migration through the endothelial junction. (5) Once in the interstium, neutrophils are exposed to a medley of chemoattractants and begin to migrate to the site of injury. (6) Neutrophils secrete a range of cytokines that participate in orchestrating the immune response. (7) Bacterial killing is achieved through phagocytosis, exposing entrapped bacteria to antimicrobial proteins and reactive oxygen species within the phagolysosome. (8) NETosis is a process by which decondensed chromatin is extruded into the extracellular space covered with an array of neutrophil-derived antimicrobial protein. (9) To migrate through dense extracellular matrices, neutrophils use granules containing high concentrations of proteases. (10) Clearance of an apoptotic neutrophil by a macrophage. Once an infection is contained, apoptosis and clearance of apoptotic neutrophils are central to the resolution of inflammation. Persistence of a proinflammatory neutrophilic response is associated with greater tissue damage. NETosis, generation of neutrophil extracellular traps.

To understand how neutrophil functions might contribute to poor outcomes in CAP requires an understanding of how these cells function optimally. Classical neutrophil functions have been extensively reviewed elsewhere (by Amulic *et al* 23), and figure 2 provides a summary of these functions. Of note, neutrophils are among the first-line effector cells to be recruited in inflammation, whether the cause of inflammation is cancer, infection or autoimmunity to kill potential pathogens or clear inflamed tissue. Neutrophils also have a role in the resolution of inflammation and angiogenesis.^24^ The importance of neutrophils in infection and inflammation is demonstrated clinically by specific congenital neutrophil deficits such as chronic granulomatous disease, which predisposes to severe, recurrent and potentially fatal infections^20^ but also by conditions where neutrophils responses are poorly contained, such as alpha 1 antitrypsin deficiency. Neutrophils have the potential to cause significant tissue damage due to their cytotoxic contents and therefore are maintained in three main states: quiescent, primed and activated.^25^ Priming is a process whereby exposure to a stimulus then increases the neutrophil response to a subsequent agonist; it is a prerequisite for tissue damage and is thought to exist as a safety mechanism to prevent unnecessary activation. The lung is a key site of neutrophil depriming, but this function is impaired in lung disease, potentially leading to the sustained activation of neutrophils.^26^

The lung has a stereotyped inflammatory response to infection regardless of aetiology, and this response includes the infiltration of neutrophils and macrophages in response to chemotactic signalling, which originates in the lung. Viruses are known to impair the host immune responses, allowing opportunistic bacteria and fungi to invade tissue. A blunted neutrophil response to these secondary infections has been implicated in the increased prevalence of CAP after influenza. In humans, influenza A infections lead to increases in local and systemic concentrations of interleukin 8 (CXCL8), causing ] neutrophil activation and recruitment, and concentrations of CXCL8 correlate with increasing disease severity.^27^ In keeping with this, neutrophils are increased in the lungs and blood after infection with pathogenic viruses in mice and humans,^28^ with cell depletion murine studies demonstrating that neutrophils are necessary for recovery from severe, but not mild, influenza A infection.^29^ In animal models, neutrophil migration to the lungs during a viral challenge appears to occur in two waves: the first wave peaks at 24 hours, and the second wave then increases over time until disease resolution or death. It is hypothesised that these waves may reflect different phenotypes of neutrophils, first, classical antimicrobial and then prorepair,^30^ with complex interactions between innate immune cells.

### Immunosenescence: impact of an ageing host on neutrophil function

Senescence or biological ageing is the gradual deterioration of functional characteristics associated with age and can refer to a whole organism (such as the ageing human), a tissue or cell. Immunosenescence is an age-associated immune dysfunction and is associated with ‘inflamm-ageing’,^14^ demonstrated by elevated levels of proinflammatory cytokines in older adults, leaving their immune system continuously activated.

Neutrophil functions also demonstrate immunosenescence. Neutrophil numbers are maintained, both in the periphery and as progenitors in the bone marrow^31^; however, older adults are more prone to neutropenia during infection as response to G-CSF appears blunted.^32^ Some studies suggest that neutrophils from older adults display altered cytokine production, but there is inconsistency as to whether this manifests as decreased or increased proinflammatory cytokines, and this might be stimuli specific. Chemotaxis is impaired in healthy older adults, demonstrating reduced accuracy of migration without impeding the ability of cells to move.^33^ Neutrophils from healthy older adults display a decreased capacity to phagocytose opsonised bacteria.^34^ Reactive oxygen species (ROS) generation and associated antimicrobial killing are impaired,^35^ although these defects are not uniform and again may be stimuli specific.^36^ Generation of neutrophil extracellular traps (NETosis) is impaired in healthy older adults and in older mice in a *S. aureus* infection model.^35 37^ Neutrophils from older donors have increased susceptibility to spontaneous and induced apoptosis and reduced capacity to prolong their lifespan.^38^ These blunted functions predispose towards infection. Of note, however, age-related neutrophil dysfunction does not appear ubiquitous or permanent. A recent study in aged cyclists has shown reduced features of immunosenescence across a number of cell types and functions,^39^ and physical activity has been shown to reduce systemic inflammation in a prospective study of older adults.^40^

### Neutrophil responses to pneumonia in older adults

During severe infections in older adults and aged mice, profound neutrophil dysfunction has been described across all effector functions. Toll-like receptor signalling (implicated in neutrophil ROS generation, cytokine production and increased survival) is decreased in older age.^41^ The accuracy of neutrophil migration is impaired in older adults with CAP, and this remains diminished for at least 6 weeks following the episode of CAP,^33^ a pattern not seen in neutrophils isolated from young adults with CAP. The role of ageing in phagocytosis remains controversial. Neutrophil phagocytosis is reduced in some^42^ but not all studies.^43^ This is likely to be related to methodological issues and may be stimuli specific. Timing is likely to be crucial, some studies looked at phagocytosis three days after onset of sepsis, and others examined soon after the onset of sepsis. Phagocytosis, especially of SP, is markedly increased by opsonisation.^44^ This antibody-mediated process can be limited at the extremes of life due to immaturity or failure to generate antibody in response to vaccination.^16 45^

Reduced NETosis has been described in older adults with CAP,^46^ but both aggravated and impaired NETosis is associated with increased mortality, supporting perhaps there being an ‘ideal’ level of NETosis, which might be stimuli specific. High levels of NETosis predict progression to ARDS and high mortality.^47^ NETosis may be less protective in older adults with CAP as both SP and ntHI have mechanisms to evade or degrade NETs.^48^ Finally, the blunted ability of neutrophils from older people to respond to survival signals might also compromise host response to CAP. The high reinfection rate following CAP in older people may also reflect immunosenescence as patients with the most dysfunctional neutrophils are at the highest risk of secondary infections.^49^

### Neutrophil function in children

Examining the impact of infection on neutrophils in children is challenging for three reasons. First, children are a very heterogeneous population with vast differences seen from preterm infants through to young adults.^50^ Second, the assays used previously required high numbers of neutrophils and thus large volumes of blood. Often in studies of infants, cord blood is used; however, cord blood is not entirely representative of peripheral blood. Third, there are ethical issues with collecting blood from healthy children. There is an established body of evidence regarding neutrophil function in preterm and term infants, and children with established diseases affecting neutrophil function, such as haematological malignancies or specific neutrophil disorders such as chronic granulomatous disease, but a paucity of data regarding neutrophil functions in healthy children who develop infection with no known neutrophil defect, despite one-third of children who develop sepsis having no pre-existing medical condition.^3^

### Neutrophil function in preterm and term infants

The fetus develops in a sterile intrauterine environment, meaning that at birth, it relies on its innate immune system; however, neutrophils from preterm infants have significant functional differences compared with those from older infants (see table 4). Neutrophils from preterm infants have functional responses which reflect that of older adults. Preterm infants have higher levels of circulating immature granulocytes and increased frequency of neutropenia due to low cell mass of neutrophils when compared with term neonates or adults.^51^ This high number of circulating immature neutrophils contributes to dysfunction, as immature neutrophils are functionally poor.^50^The low cell mass typically rises to levels seen in adults by 4 weeks of age but remains blunted if there is concurrent illness.^52^

**Table 4:** Neutrophil functions in healthy preterm and term neonates compared with healthy adult values

Neutrophil function	Preterm infant	Term infant
Migratory ability^55^	↓↓	↓
Degranulation^57^	↓↓	↓/ ↔
Phagocytosis^88^	↓↓	↔
NET generation^60^	↓↓	↓
ROS generation^53^	↔	↔

References are as given.

↔, similar ‘normal’ function; ↓, reduced function; NET, neutrophil extracellular trap; ROS, reactive oxygen species.

Preterm infants have reduced migrational capacity compared with term infants,^53^ but by 3 weeks of age, migration is comparable to that of term infants.^54^ The mechanisms underlying the impaired migration are thought to relate to altered intracellular calcium mobilisation and abnormal cytoskeletal arrangement.^55^ Degranulation has been demonstrated to be preserved in term infants compared with adults, but reduced in preterm infants^56^; conversely, other studies have shown that term infants also have reduced degranulation compared with adults.^57^ Some of the differences seen are stimuli specific, with preterm infants having normal phagocytosis to bacteria but reduced phagocytosis to *Candida* species.^53^ In stressed preterm or term infants, phagocytosis is significantly reduced to all pathogens.^50^ ROS generation is normal in preterm and term infants but reduced when these infants are under stressful conditions,^58^ and when the preterm infants remain unwell, their ability to generate ROS remains impaired at 1–2 months.^58^

Deficits in neonatal phagocytosis have been attributed to lack of maternal immunoglobulin to facilitate opsonisation.^18^ Phagocytosis is poorest in infants <33 weeks gestation and persists for 1–2 months.^18^ Administration of immunoglobulin to preterm infants restores phagocytosis^59^ in vitro; however, trials of immunoglobulin therapy in neonatal sepsis have failed to demonstrate any clinical benefit. NETosis is impaired in both term and preterm infants; this appears to not be stimuli specific, but infants are only able to generate NETs via a ROS-dependent mechanism.^60^ Apoptosis and clearance of apoptotic neutrophils are also abnormal in preterm infants, with delayed apoptosis contributing to pulmonary injury.^61^

Understanding the mechanisms underlying these observations is challenging. Certainly, the immune system of an infant needs to have significant tolerance to prevent uncontrolled activation on exposure to the maternal microbiome and environmental factors.^62^ The limited number of neutrophils recovered from infants has meant that investigating the underlying mechanisms of dysfunction is challenging. However, a recent study by Kan *et al* has investigated the dysfunction seen in monocytes from preterm infants and concluded that there are major differences in the metabolic pathways between preterm infants and adults, and this may explain the broad defects seen.^63^

It is unclear when neutrophils from children develop ‘young adult’ characteristics. Yegin *et al* identified that migration was significantly poorer in healthy children than in healthy adults,^64^ but it reached adult levels between 2 and 5 years of age. This blunting of responses coincides with the period of greatest susceptibility to infection, again linking infection susceptibility and neutrophil responses at the extremes of age.

### Proposed intracellular mechanisms associated with neutrophil dysfunction in CAP

Cytosolic calcium is crucial to many neutrophil effector functions. Impaired calcium flux has been associated with defective migration, ROS generation and degranulation,^65^ and neutrophils from older adults have higher resting calcium levels and reduced calcium flux during effector functions.^36^ Intracellular calcium flux and protein C kinases have been implicated in age-associated changes in apoptosis.^66^ The phosphoinositol 3-kinase (PI3K) signalling pathway has been implicated in neutrophil migration and the ability of neutrophils to appropriately target infectious or inflammatory signals. Modification of PI3K signalling improves migratory accuracy,^67^ and this intracellular signalling pathway is also targetable by statins through their effects on small GTPases.

Studies of aged human CAP have not assessed neutrophil function before the infective insult, so it is unclear whether the impaired effector functions seen in CAP reflect a lower baseline preceding the event or a response to CAP. However, exposing neutrophils from young adults to pooled plasma from older patients with CAP and sepsis replicates the functional deficits seen in sepsis,^33^ which suggests that the CAP environment can alter cellular function. In keeping with this, murine models of sepsis in older mice show transcriptomic changes in neutrophils which would contribute to cellular dysfunction, implicating cellular energetics and epigenetics.^68^

Neutrophils have very few mitochondria, which do not play a role in energy metabolism, and the energy required for neutrophil activity is derived from glycolysis.^69^ Effector functions such as migration and NETosis are glucose dependent, and the importance of the pentose phosphate pathway for neutrophil function is clearly observed in patients with G6PD deficiency or impairment, in which the development of infections is common due to dysfunctional neutrophil microbicidal mechanisms. There is some evidence to suggest glycolytic activity is increased with age, with an increase in pyruvate kinase activity,^70^ which would be predicted if ageing was associated with constitutive PI3K activity.

Hyperlactataemia is an independent predictor of mortality in both pneumonia and sepsis.^71^ Much evidence now supports the view that hyperlactataemia is not only due to tissue hypoxia or anaerobic glycolysis but also due to increased aerobic glycolysis (the conversion of pyruvate to lactate to generate ATP in the presence of oxygen). Neutrophils are highly glycolytic cells leading to significant lactate production, especially when activated. Intracellular lactate can influence many intracellular pathways and interacts with the glycolytic pathway, reducing glycolysis and thus energy availability to the cell.

Recent studies involving the effects of tumour-derived lactate suggest that lactate may have an immunosuppressive effect in its local environment and is an active signalling molecule in a wide range of immune cells via specific receptors.^72 73^ In vivo models support the concept that lactate may be immunosuppressive since pretreatment with lactate reduces inflammation and injury in a sterile, lipopolysaccaride-mediated hepatitis and pancreatitis model.^72^ Inhibition of glycolysis in murine models of sepsis improved survival by decreasing lactate production and cytokine production.^74^ These studies all suggest that modulation of lactate biology may have therapeutic potential to enhance immune and neutrophil functions. New studies are urgently needed to explore the possibility of using neutrophils’ glycolytic pathway to enhance the response to infection, particularly in the extremes of age.

## Neutrophil function as a therapeutic target

There is evidence to show that excessive or blunted neutrophil activity can worsen outcomes for patients, be they young or old. Neutrophils can cause significant tissue damage with proteases; this proteolytic activity is required for normal function but is also associated with tissue damage. High levels of proteases are identified in the bronchoalveolar fluid from patients with severe pneumonia^75^ and are associated with increased risk of ARDS. Proteases are also able to impair the function of the innate immune system by cleaving proteins required for activation. In animal models, inhibition of proteases leads to a reduction in inflammation and improvement in survival.^76^

Ex vivo treatment of neutrophils from patients with sepsis with G-CSF improves phagocytosis.^77^ However, clinical trials of G-CSF or GM-CSF in sepsis and or pneumonia have failed to replicate these results with no improvement in mortality^78 79^ or in specific neutrophil functions.^80^ In addition, use of G-CSF risks increasing neutrophilic inflammation; however, several large meta-analyses have not demonstrated a significant increase in adverse outcomes with G-CSF treatment.^78 79^

NETosis has also been associated with significant tissue damage. Patients with the highest levels of NETosis had increased risk of progression to ARDS and increased mortality,^47^ although low levels of NETosis has also been associated with poor outcomes.^81^ Clearance of apoptotic neutrophils and appropriate levels of apoptosis are also crucial to resolution of inflammation; delayed apoptosis and failure to clear apoptotic neutrophils are associated with increased risk of progression to ARDS and high mortality.^82^

Clearly, this suggests that there is an optimal level of neutrophil functions, and any therapy targeting neutrophils needs to normalise rather than exaggerate function to prevent excessive tissue damage, which is associated with increased mortality. Early studies have suggested this can be achieved, potentially through modification of neutrophil signalling pathways.^83^

## Conclusion

CAP can be considered as a sentinel event that signals high risk of short-term and long-term mortality and future readmission in both young children and older adults. Despite widespread use of vaccination, low prevalence of antimicrobial resistance and improvements in sepsis care, mortality and reinfection rates remain high, with survivors often left with significant impairments. Age-related changes in neutrophil function may be one of the reasons that the very young and the very old are more likely to develop CAP, and heightened dysfunctional responses during infection may be causally associated with poorer outcomes and the increased likelihood of secondary infection. Our evolving understanding of immunology has allowed us to harness the immune system to better target cancer cells, and more recently, there is building evidence that immunosenescent neutrophil dysfunction can be improved. Individual cellular functions can be modified by focusing on selective pathways, but in the face of global cellular dysfunction at the extremes of age, there may be more benefit if fundamental biological pathways are targeted, such as cellular energetics. Modifying neutrophil therapeutically remains a challenge, but perhaps now is the time to walk the tightrope Heinrich Rohrer referred to and see if we can use this highly effective phagocyte to improve patient outcomes.
